# Severe Complications of Artificial Urinary Sphincter Placement in a Young Woman With Neurogenic Urinary Incontinence: A Case Report

**DOI:** 10.7759/cureus.41097

**Published:** 2023-06-28

**Authors:** Dimitrios Diamantidis, Georgios Tsakaldimis, Stavros Lailisidis, Nikolaos Panagiotopoulos, Charalampos Kafalis, Stylianos Giannakopoulos, Christos Kalaitzis

**Affiliations:** 1 Department of Urology, University General Hospital of Alexandroupolis, Alexandroupolis, GRC

**Keywords:** neurogenic lower urinary tract dysfunction, myelomeningocele, neurogenic urinary incontinence, artificial urinary sphincter, female urinary incontinence

## Abstract

This article reports a case of a 40-year-old woman with a history of myelomeningocele and neurogenic urinary incontinence who developed erosion and fistula formation following the placement of an artificial urinary sphincter (AUS) when she was 18 years old. The patient had a long-standing history of urinary incontinence that was unresponsive to prior surgeries for meningomyelocele. She reported the loss of uro-fecal material from the vagina but did not seek further medical evaluation until the age of 40. Clinical examination revealed protruding tubes from the suprapubic region. The administration of a contrast agent through one of the two tubes led to the visualization of intestinal loops, and the administration of a contrast agent through the urethra confirmed the connection between the rectum, urinary bladder, and vagina. Due to the absence of reliable surgical history and in the absence of abdominal discomfort, bilateral nephrostomies were initially performed to prevent further uro-fecal material loss. The patient showed significant improvement, decided to not seek further evaluation and surgical treatment, and remained socially integrated during the follow-up period of 20 years. This case highlights the severe consequences of AUS placement in a young woman with neurogenic urinary incontinence and emphasizes the importance of proper patient selection and management in the presence of underlying neurological disorders.

## Introduction

Urinary incontinence is a common problem in patients with myelomeningocele, resulting from detrusor overactivity, poor bladder compliance, and sphincter dysfunction [1]. Neurogenic lower urinary tract dysfunction affects over 90% of individuals with myelomeningocele, leading to inherent damage to the lower motor neurons and impairment of urinary sphincter function. The artificial urinary sphincter (AUS) is used to treat urinary incontinence in patients with weakened or damaged sphincter muscles that are not responsive to non-surgical interventions [1-3]. However, AUS placement in female patients not only has specific indications and contraindications, particularly in those with neurogenic urinary incontinence [4,5]. It is also associated with particular challenges and controversies and is typically reserved as a last resort option after other failed treatments [6-8]. Preoperative clinical and urodynamic assessments of high quality are essential for optimal patient selection [4,9].

Complications following AUS placement are not uncommon, with mechanical malfunction, erosion, infection, and atrophy being the most frequently reported. In patients with neurogenic urinary incontinence, the rates of complications and reoperations tend to be higher as compared to non-neurogenic patient groups, highlighting the need for comprehensive patient education regarding success rates and the potential necessity for additional interventions [4,9-13].

This case report describes a patient with a neurogenic bladder who experienced erosion and fistula formation following AUS placement. The patient's reluctance to seek medical evaluation contributed to the severity of the complications. To the best of our knowledge and based on a thorough review of the available literature, no other cases similar to this have been reported.

## Case presentation

A 40-year-old woman presented to our outpatient clinic with a history of involuntary leakage of urine and fecal contents from the vagina for almost 20 years. The patient reported a childhood history of myelomeningocele and multiple surgeries for its treatment. Due to the loss of her medical records, the procedures' details were unclear. She had a long-standing history of urinary incontinence, which was not improved by prior surgeries for meningomyelocele. At the age of 18, an AUS was placed to manage her urinary incontinence without a previous urodynamic assessment. Two years after the placement of the AUS, without any prior follow-up, the patient reported the loss of uro-fecal material from the vagina without any associated feeding difficulties or abdominal pain. However, she did not seek further medical evaluation and isolated herself socially, using sanitary napkins.

**Figure 1:**
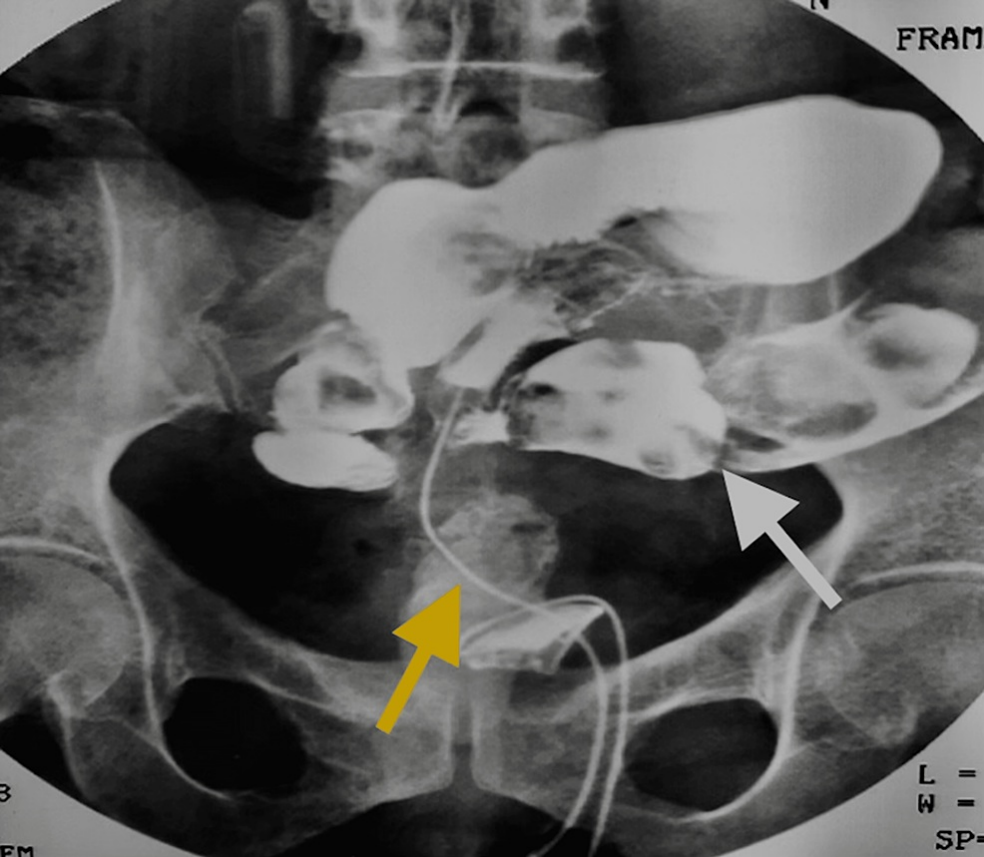
Administration of contrast agent through one of the protruded tubes from the suprapubic area (yellow arrow) revealed intestinal coils (white arrow)

**Figure 2:**
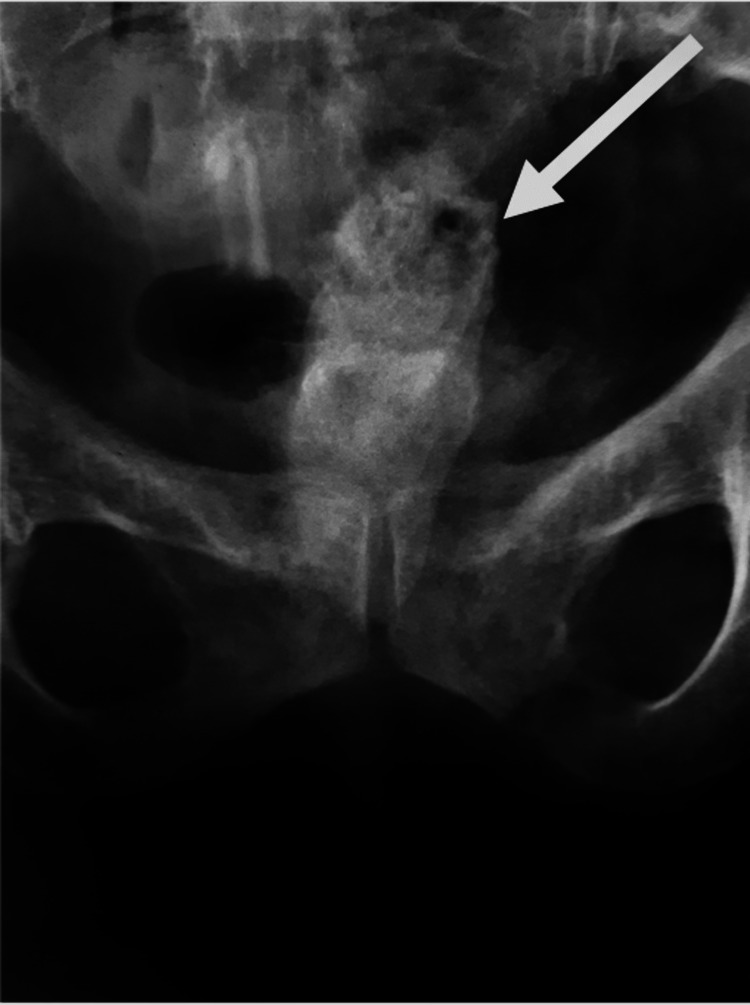
Contrast injection through the urethra during cystoscopy showed a cavity connecting the rectum and bladder (white arrow), with concurrent loss of the contrast agent from the vagina

During clinical examination, two tubes were found protruding from the suprapubic region. Administration of contrast agent through one of these tubes revealed intestinal coils (Figure 1) while during cystoscopy, entry of the instrument into an unknown space without basic anatomical structure. Contrast injection through the urethra showed a cavity connecting the rectum and bladder, with concurrent loss of the contrast agent from the vagina (Figure 2). It was hypothesized that following the implantation of the AUS, the patient exhibited one of the most severe complications associated with it, erosion. Furthermore, the patient's reluctance to seek medical assistance resulted in the erosion of neighboring anatomical structures, including the rectum, urinary bladder, and vagina, consequently leading to the development of fistulas connecting these structures.

The patient remained asymptomatic for abdominal discomfort throughout the specified duration, and her renal function exhibited normal parameters as determined during the clinical evaluation. Given the patient's history of multiple surgeries without a comprehensive surgical record, as well as the absence of urgent clinical indicators, it was determined that an emergent exploratory laparotomy would not be pursued due to the potential associated complications. Instead, bilateral nephrostomies were placed to prevent further vaginal loss of uro-fecal material. The patient reported a significant decrease in vaginal discharge and resumed defecation of formed stool within the first week after nephrostomy placement. Following the initial treatment, the patient was advised to undergo a CT scan. However, due to the prompt improvement of her symptoms, she declined further investigation. Throughout a 20-year follow-up period (with biannual nephrostomy replacements), despite the absence of surgical intervention for fistula repair, the patient consistently reports the presence of well-formed feces and the absence of any discernible uro-fecal leakage.

## Discussion

Myelomeningocele is a serious congenital neural tube defect characterized by a defect in the spinal cord and the meninges, which can result in motor and sensory deficits, bladder and bowel dysfunction, and other complications [1]. More than 90% of individuals with myelomeningocele have neurogenic lower urinary tract dysfunction [2]. Urinary incontinence is a common problem in patients with meningomyelocele, resulting from detrusor overactivity, poor compliance of the bladder, and sphincter dysfunction [1]. The cause of urinary incontinence in individuals with spina bifida is believed to be primarily due to injury to the lower section of the spinal cord and/or sacral nerve roots, which leads to inherent damage to the lower motor neurons. This impairment of neural supply endangers the function of the urinary sphincter, resulting in a deficiency of resistance in the urethra [3].

The AUS is utilized as a treatment modality for urinary incontinence in patients who exhibit weakened or damaged sphincter muscles that non-surgical interventions cannot adequately manage. Its indications encompass a broad range of conditions, comprising post-prostatectomy incontinence, neurogenic bladder dysfunction, congenital anomalies, and failed previous surgical interventions [4,5].

Nevertheless, AUS implantation has been increasingly offered to female patients who suffer from severe stress urinary incontinence caused by intrinsic sphincter deficiency; the exact role of the AUS is not well-established. However, the use of AUS is typically reserved as a final option after other treatments have proven ineffective. The European Association of Urology (EAU) indicates that the placement of an artificial urinary sphincter in women should be performed only in cases with complicated stress urinary incontinence as a last resort option and in expert centers [6,7]. On the other hand, the American Urological Association (AUA) does not mention the placement of an AUS in female patients [8]. In all cases, it is essential that a preoperative clinical and urodynamic assessment of high quality be conducted, something which was not carried out in the reported case [4,9].

The placement of AUS is not recommended in patients with low-capacity and compliance urinary bladders, recurrent urinary tract infections, urethral diverticula, complex urethral strictures, and in patients without the mental skill to operate the pump. There is a relative contraindication for certain cases and in patients with stones disease, urinary tumors, bladder neck contractures, detrusor overactivity, and high-grade vesicoureteric reflux. Additionally, some authors consider incontinence caused by radiation therapy as a contraindication to AUS implantation in female patients [4,10,11].

The placement of an AUS is accompanied by a high rate of complications, estimated at 26% [4]. The most common complications include mechanical malfunction, erosion, infection, and atrophy, which can ultimately result in recurring instances of incontinence [4,9,10]. Infection and erosion are the most severe complications that require immediate surgical intervention, leading to the removal of the device and subsequent delayed reimplantation [4]. In a series of female patients, the infection rate was 4.8% while erosion was 8.1%. Erosion included the vagina, urethra, bladder, and labia majora [11]. In patients with neurogenic urinary incontinence, it is important to note that the rates of complications and subsequent re-operations tend to be higher compared to non-neurogenic patient groups, potentially reaching up to 60%. Consequently, it is highly recommended that patients receive comprehensive and transparent information regarding success rates and the potential necessity for additional interventions [12,13].

In this particular case, the decision to forgo surgical intervention as the first-line treatment was influenced by several factors. Foremost, the potential complications associated with surgery, coupled with the non-urgent clinical condition of the patient, led to a deliberate consideration of alternative options. The medical team conducted a comprehensive risk-benefit analysis and determined that a non-surgical approach would be the most appropriate initial course of action. Additionally, it is noteworthy that the treatment modality offered to the patient subsequently became the primary approach due to the patient's expressed preference. The principles of shared decision-making and patient autonomy played a pivotal role in shaping the treatment plan. This adds an important dimension to this case and prompts a discussion surrounding the significance of patient desires and goals in treatment selection. The intriguing observation derived from the literature review was the absence of existing reports discussing the omission of surgical interventions in cases comparable to this. This underscores the novelty and rarity of this case, emphasizing the significance of documenting and discussing alternative treatment approaches, particularly when dealing with a patient who does not adhere to medical advice.

## Conclusions

Proper patient selection and management are necessary when considering AUS placement in females with neurogenic urinary incontinence. The AUS carries the risk of mechanical failure, erosion, infection, and atrophy, which can lead to recurrent incontinence. Additionally, thorough preoperative assessment and patient education about potential complications and re-operation rates are critical while patients with neurogenic urinary incontinence are particularly prone to higher complication and re-operation rates. Therefore, providing patients with accurate information about success rates and the potential need for re-interventions is crucial.
